# Knowledge, attitudes, and practices of cattle farmers regarding ticks, tick-borne diseases, and zoonotic risks in Borno State, Nigeria: A cross-sectional survey

**DOI:** 10.14202/vetworld.2025.3942-3958

**Published:** 2025-12-14

**Authors:** Samson Anjikwi Malgwi, Matthew A. Adeleke, Moses Okpeku

**Affiliations:** Discipline of Biological Sciences, School of Agriculture and Science, University of KwaZulu-Natal, Westville, Durban, South Africa

**Keywords:** acaricide use, Borno State, cattle farmers, knowledge, attitudes, and practices, Nigeria, One Health, tick-control practices, tick infestation, tick-borne diseases, zoonotic infections

## Abstract

**Background and Aim:**

Ticks and tick-borne diseases (TBDs) remain a major constraint to cattle production, responsible for substantial economic losses through reduced productivity, increased treatment costs, and high mortality. Beyond livestock impacts, ticks transmit a range of zoonotic pathogens, posing significant health risks to communities in close contact with cattle. Despite Borno State having the largest cattle population in Nigeria, there is no prior documentation of cattle farmers’ knowledge, attitudes, and practices (KAP) regarding ticks, TBDs, and their zoonotic implications. This study aimed to assess farmers’ awareness, preventive behaviors, and tick-control strategies, while evaluating the influence of formal and informal education on these variables.

**Materials and Methods:**

A cross-sectional descriptive KAP survey was conducted among 492 cattle farmers across Maiduguri Metropolitan Council and Jere Local Government Area between November 2024 and February 2025. Data were collected using a semi-structured and pre-validated questionnaire translated into local languages. Descriptive statistics summarized KAP, while Chi-square tests assessed associations between education and key outcome variables (significance level: p ≤ 0.05).

**Results:**

Most farmers (77.2%) reported observing ticks on their cattle, and 82.9% recognized their role in livestock disease transmission. Tick occurrence was highest during the rainy season (83.7%). Although awareness of livestock TBDs was high, more than half (54.4%) were unaware that ticks transmit diseases to humans. A large proportion (59.8%) reported previous tick bites, but only 10.2% sought medical care afterward. Combined control through acaricides and handpicking was the predominant practice (78.9%). Significant differences between formally and informally educated farmers were observed for lesion recognition after tick bites (χ² = 128.04; p ≤ 0.001), tick-control method (χ² = 26.30; p ≤ 0.001), frequency of handpicking (χ² = 44.27; p ≤ 0.001), and acaricide application methods (χ² = 57.45; p ≤ 0.001).

**Conclusion:**

Farmers demonstrated good knowledge of ticks and livestock TBDs but exhibited low awareness of zoonotic risks and poor health-seeking behavior following tick bites. Strengthening public health education, promoting protective practices, and integrating zoonotic TBDs into One Health policies are essential to reducing risks among high-exposure populations.

## INTRODUCTION

Livestock production, particularly cattle farming, plays a central role in Nigeria’s agricultural economy. This sector contributes approximately 15%–20% to the agricultural gross domestic product (GDP) and 5%–6% to the national GDP [1]. Borno State has the largest cattle population in the country, with over 2 million of the national total of 16 million cattle, and nearly 70% of its population depends on agriculture for their livelihood [2]. Among all livestock species, cattle remain the most economically valuable, serving as a major source of income for farmers and other actors across the value chain [3, 4] and as a vital source of animal protein in Nigeria [5]. Borno State also hosts the largest cattle market in Northeastern Nigeria, where livestock are traded and transported across the country. Its strategic location bordering Cameroon, Niger, and Chad strengthens its role as a major livestock trade hub [6]. Despite relying largely on traditional methods of cattle farming and processing, the sector continues to sustain thousands of households [7].

Ticks are regarded as the second most important group of disease vectors after mosquitoes [8], and their global importance has long been recognized for their significant impact on human and animal health [9]. Approximately 80% of the global cattle population is affected by ticks, resulting in significant economic losses due to reduced body weight, decreased milk production, and increased control costs [10]. These impacts are of considerable economic importance and directly limit cattle farmers’ productivity [11]. As obligate blood-feeding parasites, ticks require a blood meal at every life stage, and their high reproductive capacity, efficient feeding mechanisms, and protective exoskeleton contribute to their widespread distribution and resilience in harsh environments [12, 13]. More than 899 tick species have been identified, and they transmit pathogens including protozoa, bacteria, viruses, and rickettsiae [14]. Of particular medical and veterinary importance are ticks in the family Ixodidae, especially the genera *Dermacentor*, *Ixodes*, *Rhipicephalus*, and *Hyalomma* [9]. Major tick-borne diseases (TBDs) affecting cattle in the study area include anaplasmosis, babesiosis, and theileriosis, although estimating their prevalence remains difficult due to the limited use of confirmatory diagnostic tools and weak surveillance systems.

Beyond pathogen transmission, ticks also cause indirect harm through irritation, allergic reactions, blood loss (anemia), toxicosis, and, in severe cases, tick paralysis [15]. Several tick-transmitted pathogens can infect humans, causing zoonotic diseases such as babesiosis, ehrlichiosis, Lyme borreliosis, human granulocytic anaplasmosis, and Crimean–Congo hemorrhagic fever (CCHF) [16, 17]. Individuals engaged in cattle rearing are at particularly high-risk due to frequent exposure during grazing and manual tick removal, which remains a common traditional practice among pastoralists [18]. Because vaccines and prophylaxis are unavailable for most TBDs, prevention remains the most effective strategy. Recommended measures include wearing protective clothing, using appropriate footwear, applying insect repellents, and avoiding grazing areas with high tick density [19–22]. Tick-borne zoonotic pathogens are generally underdiagnosed in humans across Africa, particularly in Nigeria, where detection is more common in ticks or livestock blood than in clinical human cases [23]. Documented pathogens include *Rickettsia africae*, *Rickettsia aeschlimannii*, *Rickettsia conorii*, *Rickettsia massiliae*, *Ehrlichia ewingii*, *Ehrlichia chaffeensis*, and *Babesia divergens* [16]. To date, CCHF remains the only confirmed TBD reported in humans in Nigeria [24]. Spotted fever group (SFG) rickettsioses, caused by members of the Rickettsiae group, are of particular public health concern and are among the most frequently diagnosed febrile illnesses in travelers returning from Sub-Saharan Africa.

Zoonotic diseases collectively account for more than 2.5 billion human infections and an estimated 2.7 million deaths annually [25]. The One Health framework, which recognizes the interconnectedness of human, animal, and environmental health, is therefore essential in preventing and controlling TBDs. The World Health Organization defines One Health as an integrated, interdisciplinary strategy for addressing public health challenges at the human–animal–environment interface, emphasizing multisectoral collaboration to ensure effective research and public health programs [26]. The origins of the One Health concept date back to 1850 when Rudolf Virchow introduced the term “One Medicine” while studying the lifecycle of *Trichinella spiralis*, recognizing the zoonotic nature of diseases [27]. Over time, the One Health approach has been instrumental in outbreak response and zoonotic disease control, including rabies prevention in Sri Lanka and Bangladesh [28], and in managing outbreaks of Hendra virus, Middle-East respiratory syndrome coronavirus, Zika virus, diphtheria pertussis and tetanus, and severe acute respiratory syndrome coronavirus 2. Its core principles, unity, teamwork, and transparency, are critical for addressing emerging health threats at the human–animal–environment interface.

Ticks belonging to the families Argasidae and Ixodidae have been reported in the study area [29]. Although some progress has been made in cattle production systems, the sector in Borno State remains largely traditional, with most farmers using extensive or semi-intensive methods. Continuous year-round grazing in tick-infested environments increases farmers’ and animals’ risk of exposure and infection [30].

Despite the large cattle population in Borno State and the high-risk of tick exposure among pastoral and semi-intensive production systems, there is a notable lack of empirical data on cattle farmers’ knowledge, attitudes, and practices (KAP) regarding ticks, TBDs, and their zoonotic implications. Existing studies in Nigeria and other African regions have primarily focused on morphological identification of ticks, prevalence of tick species, and detection of tick-borne pathogens in animals, with limited emphasis on the human health dimension. Furthermore, zoonotic TBDs are severely underdiagnosed and under-reported in Nigeria due to low clinical suspicion, inadequate surveillance, and diagnostic limitations. No published work has previously evaluated the level of awareness, preventive behavior, risk perception, and tick-control practices of cattle farmers in Borno State, despite their high-exposure resulting from year-round grazing and manual tick removal practices. The influence of formal versus informal education on farmers’ knowledge and behavior regarding tick-control and zoonotic risks also remains unexplored. This gap restricts the ability of public health stakeholders, veterinarians, and One Health authorities to design targeted interventions, risk communication strategies, and community-level health education programs.

This study aimed to conduct a comprehensive assessment of cattle farmers’ KAP concerning ticks, TBDs, and associated zoonotic risks in Borno State, Nigeria. Specifically, the study sought to (i) evaluate farmers’ awareness of tick identification, seasonal abundance, and the impact of tick infestations on cattle productivity; (ii) determine their understanding of the zoonotic potential of tick-borne pathogens and their health-seeking behavior following tick bites; (iii) document current tick-control strategies and preventive practices employed in the study area; and (iv) examine the influence of educational status, formal versus informal, on farmers’ KAP outcomes. By addressing these objectives, the research aims to generate evidence-based insights to support the development of targeted public health education programs, strengthen tick-control interventions, and inform One Health strategies to reduce both animal and human health risks associated with tick exposure in Borno State.

## MATERIALS AND METHODS

### Ethical approval

Ethical approval for this study was obtained from the Borno State Research Ethics Committee, Borno State, Nigeria (Approval Ref. No. 011/2023). All procedures involving human participants were conducted in accordance with the ethical principles outlined in the Declaration of Helsinki, the Council for International Organizations of Medical Sciences (CIOMS) International Ethical Guidelines for Health-Related Research Involving Humans, and relevant national research regulations. Before data collection, the objectives, procedures, potential risks, and expected benefits of the study were clearly explained to each participant in their preferred language (Hausa, Kanuri, Fulfulde, or English).

Written informed consent was obtained from all respondents before participation. For participants who were unable to write, verbal consent was documented in the presence of an impartial witness. Participation in the study was entirely voluntary, and participants were informed of their right to withdraw at any stage without penalty. Confidentiality and anonymity were strictly maintained by removing all personal identifiers from the data collection instruments, storing questionnaires securely, and restricting data access to the research team only. Unique identification codes were used in place of names to protect participants’ identity.

To ensure responsible data handling, completed questionnaires were stored in locked cabinets and password-protected digital files accessible only to authorized research personnel. No biological samples were collected, and the study posed minimal risk since it involved only interview-based data collection. The research team ensured that sensitive information was handled respectfully and privately, especially when discussing health experiences, tick bites, or zoonotic disease concerns.

Approval from local authorities and community leaders was also obtained to facilitate culturally appropriate engagement with cattle farmers. All enumerators received mandatory training on research ethics, confidentiality, informed consent procedures, and non-coercive interviewing techniques before fieldwork commenced. Findings from the study will be responsibly disseminated to stakeholders, including local communities, veterinary authorities, and public health agencies, without compromising the anonymity or privacy of participants.

### Study period and location

**Figure 1:**
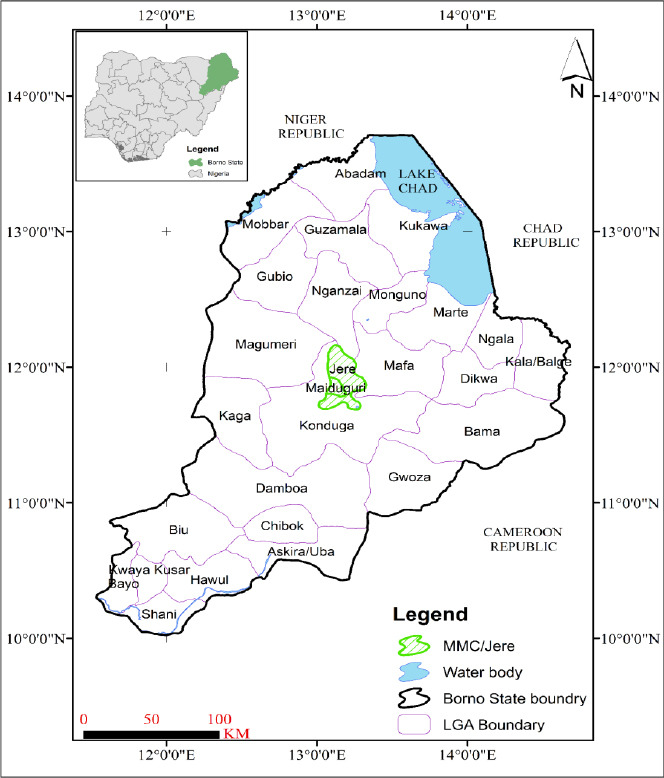
A graphical representation of Borno State, indicating the areas (highlighted in green) where the questionnaires were distributed. The areas highlighted in green represent the two local government areas where the questionnaires were administered. The blue-shaded regions indicate water bodies marked by the Lake Chad Basin. The black boundary outlines the state, which shares international borders with Cameroon, Chad, and the Niger Republic.

The study was conducted between November 2024 and February 2025 in Maiduguri Metropolis, Borno State, Nigeria. This area is situated between latitudes 11°46’ and 11°54’N and longitudes 13°06’ and 13°14’E [31]. The region is predominantly hot, with temperatures ranging from 35°C to 40°C for most of the year. Rainfall is limited, occurring mainly between July and September, with an average annual precipitation of 647 mm. The largest urban center in Northeastern Nigeria, the Maiduguri Metropolis, consists of the Maiduguri Metropolitan Council (MMC) and Jere Local Government Area (LGA) with an estimated population of 1,112,449 inhabitants [32]. It is described as roughly circular, covering an area of approximately 208 km^2^. These LGAs were selected based on their state representativeness, high livestock density (especially cattle), and relative safety. A simple random sampling technique using balloting was then employed to select wards for the respondents. The geographic coordinates of the MMC are approximately 11°50′N and 13°09′E. The Jere area falls within the latitude range of 11°40′ to 12°05′N and the longitude range of 13°05′ to 12°20′E. Figure 1 shows the study area and the distribution sites of the questionnaire.

### Study design

This cross-sectional descriptive study was conducted during the dry season. This timing was chosen primarily due to improved accessibility to farmsteads, which are mainly located outside urban areas. A standardized interview guide and semi-structured format ensured uniformity across interviews, with only the language of administration varying to accommodate participant preferences. Enumerators were recruited based on their competency and previous experience in similar surveys. The participants received additional training on research ethics and interview techniques, with further skill refinement through mock interviews and participation in the pilot study. Strict monitoring by supervisors helped ensure the resolution of field-related issues in real time. The research team implemented a standardized checklist to monitor adherence to the study protocol and conducted daily debriefings throughout data collection to ensure consistent quality assurance. The interviewers administered the questionnaires. Before the study, the personnel received professional training on administering the questionnaires effectively, ensuring unbiased responses without leading the respondents. The data cleaning process in this study involved routine field supervision to ensure strict adherence to the survey protocol and questionnaire completeness. In cases where information was missing, the respondents were re-contacted using the phone numbers provided on each questionnaire to fill in the gaps. The survey data were entered daily into an Excel spreadsheet. Incomplete questionnaires were excluded from the analysis if follow-up was unsuccessful.

### Sample size and sampling technique

A sample size of 346 participants was calculated using the Epi Info™ software version 7 designed by the Center for Disease Control to ensure statistical validity at a 95% confidence interval, allowable error of 5%, and a perception of a 10% non-response rate [33]. In the absence of available data on cattle farmers’ knowledge and attitude in the study area, a presumed prevalence of 50% was adopted for the estimation. A cross-sectional study was conducted in two LGAs of Borno State, Nigeria: MMC and Jere LGA. A semi-structured questionnaire was distributed to the participants using a convenience sampling method. This study employed a mixed or multistage sampling approach. Initially, two LGAs were selected, followed by the random selection of wards using a simple random sampling method. Respondents were then recruited within the selected wards using a convenience sampling technique. In convenience sampling, participants are chosen because they are readily accessible and available. Its main limitations lie in restricted generalizability and potential bias. However, in this study, efforts were made to mitigate these issues by diversifying data collection by employing multiple recruitment strategies and varying the data collection time throughout the day. To assess the consistency and accuracy of the data, multiple samples were collected from different groups. An increase in sample size was used to minimize the sampling error. A total of 500 questionnaires were administered within the study area to meet the sample size requirements and further increase the reliability of the survey results.

### Eligibility criteria

To be eligible for inclusion in the study, participants had to be cattle farmers and at least 18 years old at the time of data collection and reside within the study area. Participants were excluded from the study if they were under the age of 18, did not reside in the selected communities (MMC and Jere), were not directly involved in livestock rearing, were unable to communicate in the study languages (Hausa, Kanuri, Fulfulde, or English), had cognitive impairments affecting their ability to provide informed consent, or declined to participate.

### Questionnaire design and validation

The questionnaire is composed of 28 carefully planned questions, each addressing a core objective of the research. The questionnaire was adapted from a validated KAP survey [30] and further reviewed by a multidisciplinary panel including epidemiologists, veterinarians, extension workers, health personnel, and cattle farming community leaders to ensure that it was culturally appropriate and met the study’s objectives. Section A focused on the respondents’ sociodemographic characteristics, including age, LGA, herd size, management system, level of education, and years of experience. All responses in this section were categorized, allowing respondents to choose the most appropriate option. Section B comprised 9 questions focused on assessing the respondent’s knowledge of ticks and TBDs. Responses were categorized for ease of selection, as in Section A. Section C examined the attitudes of the respondents toward ticks and TBDs, containing 7 questions with structured, categorical responses. Section D emphasized the practices of cattle farmers in controlling ticks and TBDs, also comprising seven questions with predefined response options.

Experts in epidemiology and representative end users reviewed the questionnaire for face and content validity, focusing on the accuracy of its KAP sections. The review provided constructive feedback, and the corresponding corrections were applied. Post-review, the questionnaire underwent pilot testing with a group of 30 participants to assess its clarity, validity, and reliability. All participants received a clear explanation of the study’s objectives before providing their written informed consent. The pilot study data were analyzed to assess internal reliability using Cronbach’s alpha, resulting in a moderately high value (α = 0.65). Participation was voluntary, and respondents were free to withdraw at any time during the pilot and main study.

### Translation and cultural adaptation

The initial questionnaire was drafted in English. However, due to the multilingual nature of the study area, trained translators translated it into the major local languages Kanuri, Hausa, and Fulfulde. The translated questionnaires were reviewed and subsequently returned to the multisectoral team of experts to ensure that the translations accurately reflected the original meaning without altering the questions or introducing bias. Community leaders and representatives of cattle farmers were involved in the process to ensure the questionnaire was accurately translated and culturally adapted to reflect local values and perspectives. Including community members in the process was essential for maintaining ethical standards and preventing potential misinterpretations that could undermine the study’s goals. Two bilingual professionals conducted the forward and backward translations. A panel of experts reviewed the translations to ensure that the questionnaire’s clarity and contextual relevance were retained.

### Statistical analysis

After completion, questionnaires were collected from the respondents and manually checked and filtered to ensure clarity. A structured data entry protocol was adopted to ensure accuracy and consistency. Data were entered independently by two trained personnel (double entry) into Microsoft Excel version 16.0 (2025) (Microsoft, Washington, USA). A reconciliation process was then performed to resolve any inconsistencies between the two datasets with reference to the original survey instruments. Some responses were converted into percentages (%) to represent their frequency among all respondents. Associations and differences between educational status, demographic characteristics, and KAP were determined using the Chi-square (χ^2^) test. The significance level was set at 0.05; p-values below this indicate statistical significance (p ≤ 0.05). Each respondent appeared in the contingency table only once, maintaining the independence of observations. The analysis was limited to categorical variables, as required for the Chi-square test. All statistical computations, including Chi-square tests, were performed using the Statistical Package for the Social Sciences 26.0 (IBM Corp, Chicago, IL, USA). Data were presented using charts generated in Microsoft Excel (version 16.0 (2025).

## RESULTS

### Questionnaire distribution and response rate

A total of 500 questionnaires were distributed to respondents, and 492 were returned, yielding a refusal rate of 1.6%. The data collected from the returned questionnaires were used in the analysis to further improve the reliability and precision of the study, even though the sample size exceeded the estimated sample size. Since all the survey respondents were male, gender was not considered in the demographic analysis. Figure 2 presents a comprehensive overview of the respondents’ sociodemographic characteristics, including age, LGA, herd size, management system, years of experience, and educational level. These factors provide valuable insight into the demographic composition of the study respondents.

### Sociodemographic characteristics of respondents

The data from the study indicate that the largest age group among cattle farmers is the youth (18–25 years), comprising 318 respondents (64.3%), while the older group (26 years and above) accounts for 174 respondents (35.4%), as displayed in Figure 2. The MMC recorded the highest number of cattle farmers, with 272 (55.3%), compared to Jere LGA, which had 220 (44.7%). Regarding herd size, the category of 51–100 cattle had the highest number of respondents, totaling 310 (63.0%), followed by the 10–50 category with 70 respondents (14.3%). The smallest group was those owning 101 or more cattle, with 56 (22.7%) respondents.


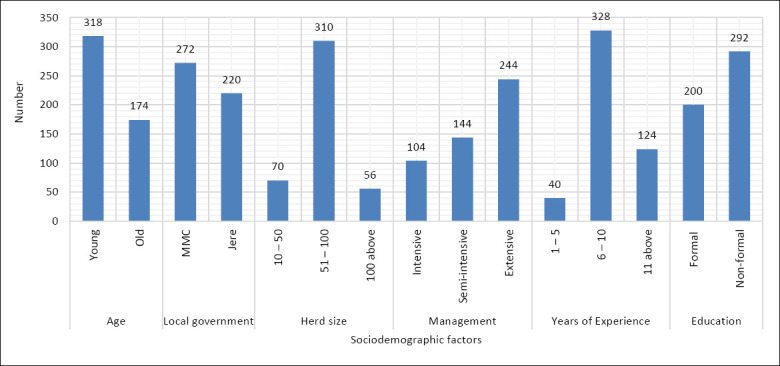


The extensive system of cattle management was the most practiced, reported by 244 respondents (46.9%), followed by the semi-intensive system with 144 respondents (29.3%), while the intensive system was the least practiced, with 104 respondents (21.1%). Regarding farming experience, the majority of the 328 respondents (66.7%) had 6–10 years of experience. Those with 11 years or more accounted for 124 respondents (25.2%), while only 40 (8.1%) had 1–5 years of experience. Educationally, 292 respondents (59.3%) had received informal education, while 200 (40.7%) had received formal education.

### KAP of cattle farmers

In this study, 380 (77.2%) respondents reported seeing ticks on their cattle, with 200 (40.7%) indicating that young animals had the highest tick prevalence, underscoring the widespread presence of ticks in the study area (Table 1). In addition, 270 (54.9%) respondents reported observing colored ticks on their animals, compared with 222 (45.1%) who reported non-colored ticks. Most respondents, 412 (83.7%), reported an increase in tick populations during the rainy season, reflecting seasonal abundance.

**Table 1:** General overview of cattle farmers’ knowledge, attitudes, and practices regarding ticks and tick-borne diseases in Borno State, Nigeria.

S. No.	Variables	Number (%)
**Knowledge**		
1.	Have you ever seen ticks on your cattle?	
	Yes	380 (77.2)
	No	112 (22.8)
2.	Ticks are mostly found in what age category of cattle?	
	Young	200 (40.7)
	Adult	292 (59.3)
3.	Do ticks transmit diseases to animals?	
	Yes	408 (82.9)
	No	84 (17.1)
4.	What type of ticks have you seen?	
	Colored	270 (54.9)
	Not colored	222 (45.1)
5.	What season of the year do you see a high of ticks?	
	Rainy	412 (83.7)
	Dry	80 (16.3)
6.	Do tick-borne diseases affect cattle production?	
	Yes	376 (76.4)
	No	116 (23.6)
7.	Have your animals been sick due to tick-borne infection disease?	
	Yes	430 (87.3)
	No	62 (12.7)
8.	Do ticks transmit diseases to humans?	
	Yes	176 (35.8)
	No	268 (54.4)
	I don’t know	48 (09.8)
9.	Do you know of any zoonotic tick-borne disease?	
	Yes	168 (34.1)
	No	324 (65.9)
**Attitude**		
11.	Have you been bitten by ticks?	
	Yes	294 (59.8)
	No	198 (40.2)
12.	What symptom did you or someone notice after being bitten by ticks?	
	Rash	166 (35.8)
	Swelling	192 (39.0)
	Nothing	134 (27.2)
13.	Have you or someone you know ever fallen sick after exposure to tick bites (fever)?	
	Yes	128 (26.0)
	No	364 (74.0)
14.	Have you ever visited a hospital after a tick bite?	
	Yes	50 (10.20
	No	442 (89.8)
15.	Are you afraid of a tick bite?	
	Yes	210 (42.6)
	No	282 (57.4)
16.	Do you protect yourself from tick bites?	
	Yes	400 (81.3)
	No	92 (18.7)
17.	Do you report ticks to a veterinarian?	
	Yes	336 (68.3)
	No	156 (31.7)
**Practices**		
18.	What method do you use to control ticks?	
	Acaricides	58 (11.8)
	Handpicking	46 (09.3)
	Both	388 (78.9)
19.	How often do you remove ticks when handpicking is used?	
	Daily	60 (12.2)
	Weekly	148 (30.1)
	Monthly	284 (57.7)
20.	If you use a chemical, how do you apply it?	
	Dips	54 (11.0)
	Spray	218 (44.3)
2.	Injection	220 (44.7)
21.	What is the frequency of acaricide application on your farm?	
	2–4 weeks	68 (13.8)

The survey further revealed that 376 (76.4%) respondents acknowledged the negative impact of TBDs on cattle productivity, while 430 (87.3%) reported that their cattle had been infected by such diseases. Regarding zoonotic awareness, 268 (54.4%) respondents were aware of the role of ticks in transmitting diseases to humans, and 260 (52.8%) were familiar with at least one zoonotic TBD. Furthermore, 60 (12.2%) respondents reported knowing someone who had been hospitalized due to a tick-borne illness (Table 1).

Regarding the attitudes of the respondents, 298 (59.8%) reported having been bitten by ticks. The most common symptoms following a tick bite were swelling at the bite site, which was reported by 192 (39.0%) respondents, and rash, which was reported by 166 (35.8%) respondents. No symptoms at the bite site were noted by 134 (27.2%) respondents. Tick bites led to illness (typically fever) in 128 (26.0%) respondents, yet only 50 (10.2%) individuals sought medical attention at a hospital.

Interestingly, a significant proportion of the 282 (57.4%) respondents reported not worrying about tick bite risk. However, 400 (81.3%) patients took protective measures against tick bites, and 336 (68.3%) reported cases of tick infestation to a veterinarian.

The most common method used to control ticks in the study area involves a combination of manual removal (handpicking) and chemicals, as reported by 338 (78.9%) respondents. Handpicking is typically performed monthly by 284 (57.7%) respondents, while chemical control using acaricides is applied every 3 months by 166 (33.7%) respondents. Regarding the mode of application of synthetic pyrethroids, a significant proportion of respondents administered these agents by injection (220, 44.7%), followed by sprays (218, 44.3%) and dips (54, 11.0%), the least common method. Most respondents (286, 58.1%) reported using a single chemical product as an acaricide. In addition, 466 (94.7%) respondents reported carefully inspecting their clothing and body after handling tick-infested animals.

### Comparative analysis based on educational status

Table 2 shows the results of the analysis, which showed no significant differences across all sociodemographic factors, including age, LGA, herd size, management system, and years of experience. Similarly, no significant difference in knowledge levels was observed among respondents across the study area. Knowledge variables assessed included awareness of tick presence on cattle, the age category of animals most infested, knowledge of disease transmission to humans, and identification of zoonotic pathogens.

**Table 2:** Comparative analysis of knowledge, attitudes, and practices of cattle farmers regarding tick-borne diseases and their zoonotic implications in relation to formal and informal education.

Sociodemographic factors	Education			Chi-square	p-value

	Total	Formal n = 200	Informal n = 292		
Age					
Young	318	138	180	2.497	0.1140
Old	174	62	112		
Local government					
Maiduguri Metropolitan Council	272	120	152	2.718	0.0992
Jere	220	80	140		
Herd size					
10–50	70	60	10	179.19	0.0001
51–100	310	56	254		
100 above	112	84	28		
Management system					
Intensive	104	76	28	190.19	0.0001
Semi-intensive	144	100	44		
Extensive	244	24	220		
Years of experience					
1–5	40	12	28	14.50	0.0007
6–10	328	120	208		
11 above	124	68	56		
**Knowledge**					
Have you ever seen ticks on your cattle?					
Yes	380	196	184	80.66	0.0001
No	112	4	108	
Ticks are mostly found in what age category of cattle?					
Young	200	74	126	1.615	0.2038
Adult	292	126	166		
Do ticks transmit diseases to animals?					
Yes	408	194	214	45.48	0.0001
No	84	6	78		
What type of ticks have you seen?					
Colored	270	40	230	163.19	0.0001
Not colored	222	160	62		
What season of the year do you see a high number of ticks?					
Rainy	412	130	282	84.61	0.0001
Dry	80	70	10		
Do tick-borne diseases affect cattle production?					
Yes	376	200	176	101.77	0.0001
No	116	0	116		
Have your animals been sickened by tick-borne disease?					
Yes	430	188	242	84.61	0.0001
No	62	12	50		
Do ticks transmit diseases to humans?					
Yes	176	10	166	183.51	0.0001
No	268	182	86		
I don’t know	48	8	40		
Do you know of any zoonotic tick-borne disease?					
Yes	168	34	134	42.78	0.0001
No	324	166	158		
Attitude					
Have you been bitten by ticks?					
Yes	294	16	278	371.75	0.0001
No	198	184	14		
What symptom did you or someone notice after being bitten by ticks?					
Rash	166	48	118	128.04	0.001*
Swelling	192	136	56		
Nothing	134	16	118		
Have you or someone you know ever fallen sick after exposure to tick bites (fever)?					
Yes	128	60	68	2.441	0.1182
No	364	140	224		
Have you ever visited a hospital after a tick bite?					
Yes	50	40	10	33.92	0.0001
No	442	160	282		
Are you afraid of a tick bite?					
Yes	210	156	52	169.38	0.0001
No	282	44	238		
Do you protect yourself from tick bites?					
Yes	400	184	216	24.20	0.0001
No	92	16	76		
Do you report ticks to a veterinarian?					
Yes	336	174	162	53.01	0.0001
No	156	26	130		
Practices					
What method do you use to control ticks?					
Acaricides	58	14	44	26.30	0.001*
Handpicking	46	6	40		
Both	388	180	208		
How often do you remove ticks when handpicking is used?					
Daily	60	6	54	44.27	0.001*
Weekly	148	46	102		
Monthly	284	148	136		
If you use a chemical, how do you apply it?					
Dips	54	10	44	57.45	0.001*
Spray	218	60	158		
What is the frequency of acaricide application on your farm?					
2–4 weeks	68	46	22	153.97	0.001*
4–8 weeks	166	106	60		
Over 3 months	258	48	210		
Do you check your body for ticks after handling tick-infested cattle?					
Yes	466	180	286	0.0003	0.9866
No	26	10	6		
Do you use one or more acaricides to control ticks?					
One chemical	286	134	152	10.29	0.0013

Several attitude-related variables also showed no significant differences. These included being bitten by ticks, falling ill after a tick bite, seeking medical attention following a tick bite, fear of tick bites, protective behavior against ticks, and reporting tick infestation in animals to a veterinarian. However, a significant difference was observed in the type of lesion after a tick bite (χ^2^ = 128.04, p ≤ 0.001), which was associated with respondents’ level of education.

**Figure 3:**
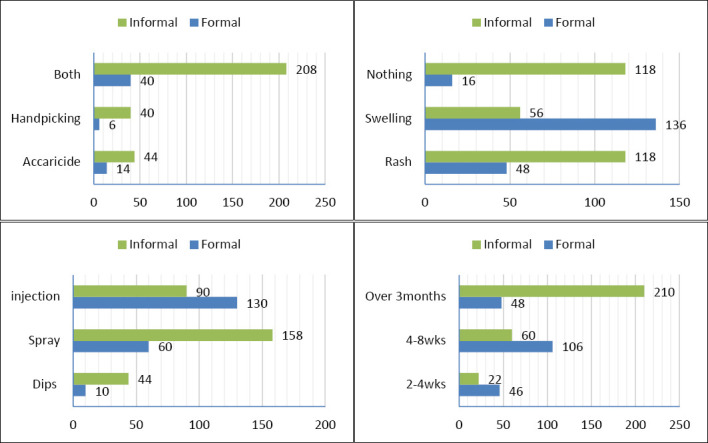
*Effect of formal and informal education on cattle farmers’ knowledge, attitude, and practice related to ticks and tick-borne diseases*. (a) Tick-control method. (b) Lesions at bite site. (c) Acaricide application method. (d) Frequency of acaricide application.

No significant differences were found for some variables, such as checking the body after handling tick-infested animals and using single or combined chemical acaricides. However, other practices varied significantly by educational status. These included the methods used to control ticks (χ^2^ = 26.30, p ≤ 0.001), frequency of handpicking (χ^2^ = 44.27, p ≤ 0.001), and acaricide application (χ^2^ = 57.45, p ≤ 0.001) (Figure 3).

## DISCUSSION

### Overview of TBDs and study rationale

The devastating impact of ticks and TBDs has been reported in both livestock and humans, leading to significant economic losses worldwide [34]. This questionnaire-based study examined the KAP of cattle farmers regarding ticks, tick bites, control measures, and their impact on human health. Despite global reports highlighting the prevalence of TBDs in both animals and humans, the human aspect of these diseases receives limited attention in many developing countries, including Nigeria. These diseases are often underestimated, with numerous undiagnosed and unreported cases [35]. In many medical facilities in Nigeria and other developing nations, TBDs are seldom considered possible causes of febrile illnesses or included in the differential diagnosis for endemic diseases such as malaria and typhoid [16]. This study aimed to address this gap despite the existing challenges.

### Sociodemographic characteristics of respondents

All respondents in the study were male, which may be attributed to cattle rearing being traditionally male-dominated in Nigeria [36]. The male dominance in cattle farming in Nigeria is primarily attributed to cultural and traditional norms, especially among the Hausa/Fulani communities, which play a central role in livestock production [37]. Furthermore, this may be attributed to the physically demanding nature of cattle farming, which is often considered unsuitable for women, particularly given the nomadic or extensive nature of the predominant farming system. Several studies have previously reported male dominance in cattle farming across various African countries, including Tanzania [38], Zimbabwe [39], South Africa [40], and Nigeria [41]. This trend is often linked to the cultural belief that men are better equipped to handle the physical and managerial challenges associated with feeding, handling, and managing livestock [40, 42, 43].

Youth reliance on cattle farming as a principal source of income was a key finding of this study. This contrasts with findings from previous studies by Chenyambuga *et al*. [44], Scholtz *et al*. [45], and Nnabuife *et al*. [46], which reported that most cattle farmers were aged >50 years. This variation may be due to the urban nature of the study area, in contrast to the rural settings of studies that recorded older farmers. Younger individuals often migrate from rural to urban areas in search of better opportunities, which may explain their greater involvement in cattle farming in this context. The study area’s youth predominance suggests a promising future for the agricultural sector, particularly cattle farming, as it reflects a shift in farmer demographics and a move away from outdated traditional practices. Young cattle farmers are more inclined to adopt technology, which acts as a catalyst for positive transformation in the agricultural sector. This development will strengthen cattle farming management, paving the way for higher productivity. The active involvement of youth in cattle farming promotes sustainability and continuity, safeguarding the future of the agricultural sector.

### Tick infestation patterns and seasonal abundance

Tick abundance during the rainy season has been reported in Nigeria, Ethiopia, and Tanzania [47–49]. This study supports those findings, as most respondents reported a high tick prevalence during the rainy season. Several factors, including rapid vegetation growth, increased humidity, favorable breeding conditions, and heightened livestock activity, have been associated with this seasonal increase, particularly in semi-intensive and extensive management systems [50–52]. The rainy season creates an environment conducive to tick reproduction and survival, thereby increasing the availability of definitive hosts and tick prevalence.

The reduced efficacy of control measures is an important factor contributing to tick abundance. Flooding during the rainy season often makes certain areas inaccessible, hindering timely animal treatment. In addition, topical acaricides are frequently washed off by rain, reducing their efficacy and necessitating repeated applications, thereby increasing livestock production costs [53]. The manual removal of ticks also becomes more difficult due to the slippery condition of wet animal skin.

### Tick species identification and public health risk

To identify the most prevalent tick species in the study area, ornamentation (coloration) was used as a distinguishing feature to differentiate between ornate (colored) and non-ornate (non-colored) ticks. Many respondents reported observing colored ticks on their cattle more frequently than non-colored ticks. This finding aligns with the climatic characteristics of Borno State, which is characterized by consistently high temperatures throughout the year. Due to their resilient cuticle structure, hard ticks have been reported as the region’s most widely distributed tick type, as they are better adapted to withstand high temperatures than soft ticks [54].

Several studies have documented the presence of *Boophilus* spp., *Dermacentor* spp., *Amblyomma* spp., and *Hyalomma* spp., with *Amblyomma* spp. being the most common in Borno State. According to Opara and Ezeh [54], the prevalence of *Amblyomma* spp. exceeds 50% in both Borno and Yobe States. *Amblyomma variegatum*, an ornate (colored) tick species, is a significant vector of TBD transmission to both animals and humans. It transmits *R. africae*, a member of the SFG, to humans [55]. This represents a serious public health risk, particularly in areas where tick-control is primarily through manual removal and awareness of tick-borne zoonoses remains low.

### TBD burden in cattle

Respondents overwhelmingly reported observing ticks on their cattle and that these animals had been diagnosed with or infected by TBDs. These diseases have contributed to declining animal productivity, particularly when tick prevention and control strategies were poorly implemented. According to the study, adult cattle exhibited a higher tick prevalence than young calves. Adult cattle are likely to have more ticks attached to their bodies because they spend more time on pastures, and their larger surface area provides more opportunities for attachment [56].

However, although the prevalence of ticks is typically higher in adults, they are more resilient to tick-borne infections. Adult cattle also have a more developed immune system, which can mount an effective response to these infections and often act as carriers. Nevertheless, severe infestations can still lead to serious health problems. In contrast, young animals are particularly vulnerable to tick-borne infections, and heavy infestations can often be fatal [57].

### Current tick-control practices and challenges

Tick-control measures involve the use of acaricides in sprays, dips, and pour-ons. Some limitations of acaricide use include their toxicity, which can result in animal product residues and environmental pollution [58]. Cost is a major factor influencing the choice of control measures. This explains why respondents with limited resources often prefer manual tick removal, known as handpicking, which is commonly practiced in Nigeria [47]. The majority of respondents reported using a combination of acaricides and handpicking as their tick-control method, with acaricides typically applied every 3 months. Manual removal was identified as the most commonly employed method of tick-control in this study.

In the study, farmers predominantly relied on a single chemical for tick-control rather than using multiple acaricides in combination, which is important to avoid the development of chemical resistance when a single chemical is used consistently over a long period. The cost of purchasing acaricides has limited respondents’ use to a single chemical agent. Respondents often combine a chemical agent with handpicking to minimize costs.

### Zoonotic awareness and educational influence

Regarding the zoonotic implications for human health, most respondents were unaware that ticks could transmit diseases to humans and could not identify any zoonotic tick-borne pathogens. These findings are consistent with those of Ndhlovu and Masika [59] in Zimbabwe and Nnabuife *et al*. [46] in Nigeria. A concerning observation is that over half of the respondents reported having been bitten by ticks. Among these, more than 70% experienced symptoms at the bite site, including rashes and swelling.

The low level of knowledge about tick-borne pathogens among respondents is likely linked to respondents’ educational level, particularly in areas where informal education is predominant. However, some respondents still lack awareness of zoonotic TBDs despite having undergone formal education. Multiple underlying factors contribute to the limited awareness observed in respondents with formal education. Most respondents who received a formal education completed only secondary school, with no advancement into tertiary institutions. Even at advanced levels, formal education, especially to the tertiary level, lacks in-depth coverage of tick-borne infestation and zoonotic diseases, unless the learner is engaged in specialized fields such as public health, epidemiology, or biological sciences.

Furthermore, a lack of integration between human and animal health strategies makes tick-borne zoonotic diseases a low priority compared with more immediate health threats, especially in areas where cases are seldom diagnosed. Nnabuife *et al*. [46] reported low awareness of zoonotic diseases in Jos, Nigeria. In contrast, Ndeerah *et al*. [60] found that respondents in Kenya had sufficient knowledge of zoonotic tick-borne pathogens and could identify several of these diseases.

In developing countries, such as Nigeria, the threat of zoonotic diseases is frequently underestimated, mainly due to inadequate surveillance and limited epidemiological data [61]. Typically, national health policies prioritize zoonotic diseases only when they attract global concern or reach epidemic status. Due to the absence of large-scale outbreaks, zoonotic tick-borne pathogens have attracted little attention from the health sector, resulting in inadequate public education initiatives.

The low awareness of the disease has also been attributed to multiple factors, such as its often mild or non-specific symptoms, which complicate diagnosis, and insufficient involvement of the public sector [62]. Sociocultural beliefs that attribute illness to spiritual or ancestral causes undermine awareness of zoonotic risks. Moreover, the close cohabitation with ticks has led to their normalization as non-threatening. The disconnection between human and animal health systems is a significant barrier to effective disease monitoring, surveillance, and public health initiatives.

### Human behavior and exposure risk

A previous study by Ndeereh *et al*. [60] has highlighted human behavior as a critical factor in the transmission of TBDs to humans, especially in areas with high infection rates and dense populations. Although most respondents reported experiencing lesions at the bite site and fever following tick bites, both of which are nonspecific symptoms commonly associated with TBD. Additional symptoms, such as nausea and vomiting, are frequently observed in humans infected with tick-borne pathogens [63].

In our study, a significant percentage of respondents reported not wearing protective clothing when grazing with their animals, regardless of the season. This, combined with reports that most respondents have experienced tick bites, which are potential vectors of zoonotic pathogens, raises serious public health concerns. Alarmingly, only 10.2% of respondents reported visiting a medical facility to seek medical attention despite noticing a lesion at the bite site or experiencing a fever. This lack of medical follow-up poses a serious public health risk, as infections may circulate unnoticed and untreated in the study area.

Personal protective equipment (PPE) and prompt body checks, especially in high-risk or tick-dense areas, have been recommended as effective control measures [20, 64]. There is growing concern that these diseases, particularly those caused by SFG rickettsiae, may be circulating in the study area. This concern is heightened by the low level of awareness and inadequate diagnostic facilities in veterinary and human healthcare centers. These infections may contribute to the commonly reported cases of fever of unknown origin in many medical facilities in the study area.

### Risk factors and educational influence on practices

The study also identified a major predisposing factor for zoonotic infections, in addition to the general lack of awareness of tick-borne zoonoses. Some respondents shared their living spaces with animals, which significantly increased the risk of zoonotic infections. This observation aligns with the findings of studies conducted in Zimbabwe [65], South Africa [40], and Nigeria [46]. Beaujean *et al*. [66] reported that respondents’ educational level is a potential risk factor for infection. Their report stated that inadequate knowledge undermines awareness of the dangers of zoonotic TBDs and their implications for human health.

The study revealed that most respondents in the area had attained only informal education, which may have influenced their level of health awareness. This suggests a high probability that many respondents may be unknowingly exposed to infection. Regarding respondents’ educational background, no statistically significant differences were observed across sociodemographic factors or knowledge. However, significant differences were found in some of their attitudes and practices, especially in the surveyed population’s ability to accurately identify and clinically describe symptoms at the site of a tick bite. In addition, formal education plays a key role in influencing the choice of effective tick-control measures.

These findings highlight the importance of education in promoting proper health practices. Therefore, a public health awareness campaign is crucial to educate the community about the dangers of zoonotic infections and preventive measures, with particular emphasis on the risk factors involved in infection transmission.

## CONCLUSION

This study provides comprehensive insight into the KAP of cattle farmers in MMC and Jere LGA regarding ticks, TBDs, and their zoonotic implications. The high response rate (492/500; 98.4%) and strong representation of youth (64.3%) reflect the active involvement of younger farmers in cattle production. Most respondents (77.2%) reported seeing ticks on their cattle, and a substantial proportion (83.7%) observed a marked increase in tick abundance during the rainy season. Although awareness of tick infestation and its negative impact on cattle productivity was generally high, knowledge of zoonotic risks was considerably lower, with only 54.4% recognizing that ticks can transmit diseases to humans. Moreover, despite 59.8% of respondents experiencing tick bites, only 10.2% sought medical care, underscoring a critical gap in health-seeking behavior.

Practices related to tick-control varied significantly by educational status, particularly in acaricide application methods (χ^2^ = 57.45; p ≤ 0.001) and the frequency of manual removal (χ^2^ = 44.27; p ≤ 0.001). The most common control method, a combination of handpicking and chemical acaricides, reflects both financial constraints and traditional farming practices. These findings emphasize the need to strengthen community-level preventive strategies, improve acaricide use practices, and promote knowledge of zoonotic TBDs.

The study identifies clear entry points for public health and veterinary interventions. Strengthening farmer awareness of zoonotic TBDs, promoting protective behaviors such as PPE use, and improving access to veterinary extension services are essential for reducing transmission risks. Educating farmers on appropriate acaricide use and resistance prevention can enhance tick-control outcomes and livestock productivity. Integrating zoonotic TBDs into routine differential diagnosis in medical facilities can improve early detection of rickettsial and other vector-borne infections.

A major strength of this study is its large sample size, high response rate, and rigorous multistage sampling approach. The use of validated questionnaires, culturally adapted translations, and double-entry data verification enhanced the reliability of findings. The comparative analysis based on formal versus informal education adds depth to understanding behavioral and knowledge-based disparities.

The study’s main limitation stems from security challenges in Borno State, which restricted access to certain high-risk pastoral regions. Convenience sampling at the respondent-selection stage may limit full generalizability. The reliance on self-reported data may also introduce recall bias, especially regarding past tick bites and illnesses.

Future studies should incorporate entomological surveillance to identify circulating tick species and pathogens, particularly SFG rickettsiae, *Ehrlichia* spp., and *Babesia* spp. Clinical studies evaluating febrile cases for TBDs would fill diagnostic gaps. Integrating molecular diagnostics and One Health surveillance systems will strengthen early detection of zoonotic TBDs. In addition, interventions focused on farmer training, improving acaricide stewardship, and assessing environmental risk factors will further contribute to sustainable TBD prevention.

Overall, the study highlights a substantial burden of tick exposure among cattle farmers and a concerning gap in the awareness of zoonotic risks. Enhancing farmer education, strengthening veterinary-public health collaboration, and implementing targeted One Health interventions are critical steps toward mitigating the impact of ticks and TBDs on both livestock and human health in Borno State. Addressing these gaps will support healthier communities, more resilient livestock systems, and improved public health outcomes.

## DATA AVAILABILITY

The supplementary data can be made available from the corresponding author upon request.

## AUTHORS’ CONTRIBUTIONS

SAM: Conceptualization, investigation, methodology, writing-original draft, software, formal analysis, and data curation. MAA: Supervision, writing-review and editing, and validation. MO: Conceptualization, supervision, writing-review and editing, validation, and project administration. All authors have read and approved the final version of the manuscript.
